# Supplementary material for: Evaluation of Ground Pressure, Bearing Capacity, and Sinkage in Rigid-Flexible Tracked Vehicles on Characterized Terrain in Laboratory Conditions
Source: Sensors (Basel). 2024 Mar 10;24(6):1779. doi: 10.3390/s24061779 (PMC10975934; doi:10.3390/s24061779)
Supplement: Supplementary file 1 [file sensors-24-01779-s001.zip › sensors-2875455-supplementary.pdf]

Supplementary Information

Pressure sensor

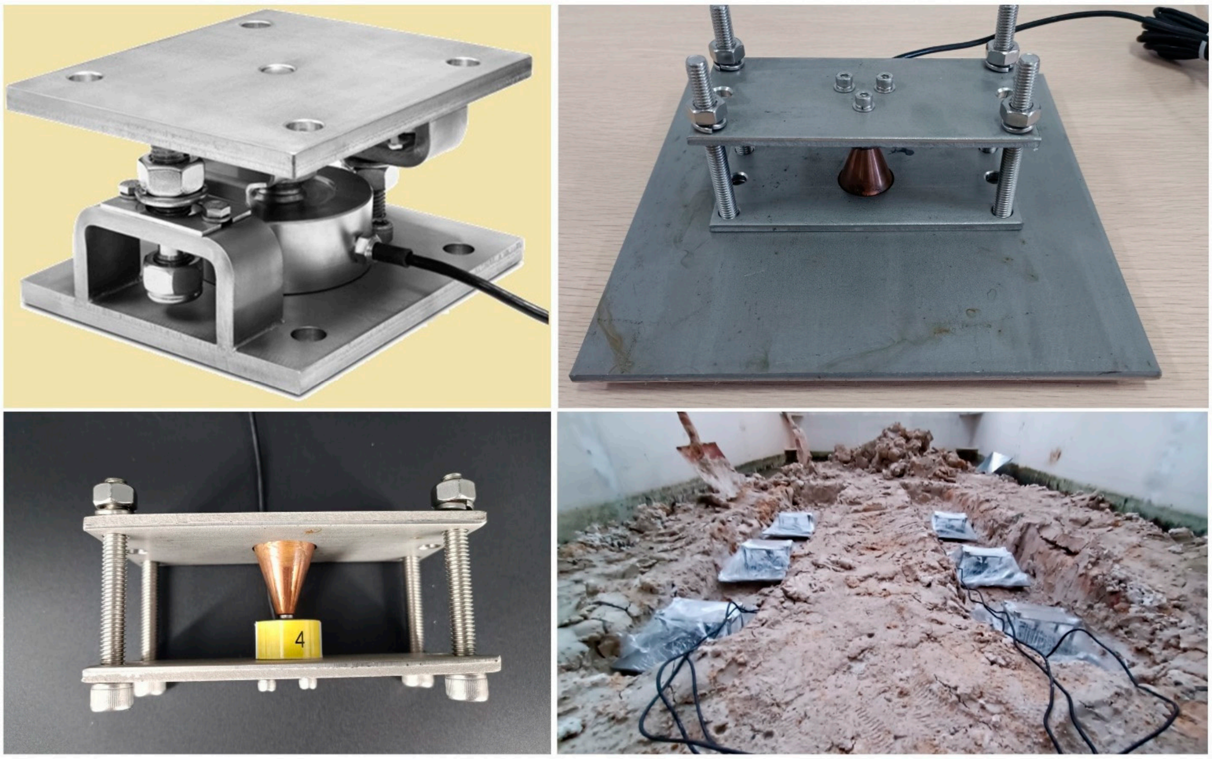

Supplementary Figure S1. Pressure sensor, including sensor bracket.

Soil bin

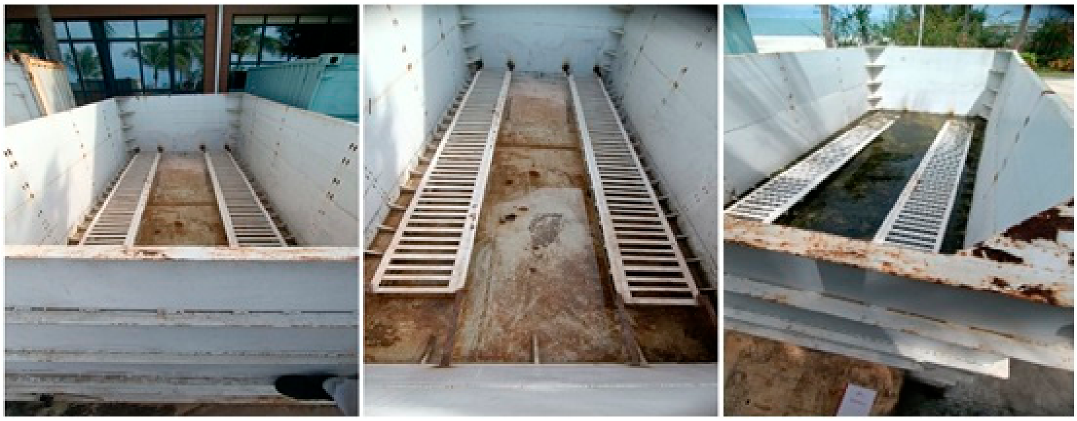

Supplementary Figure S2. Soil bin setup for the experiment.

9-axis attitude sensor

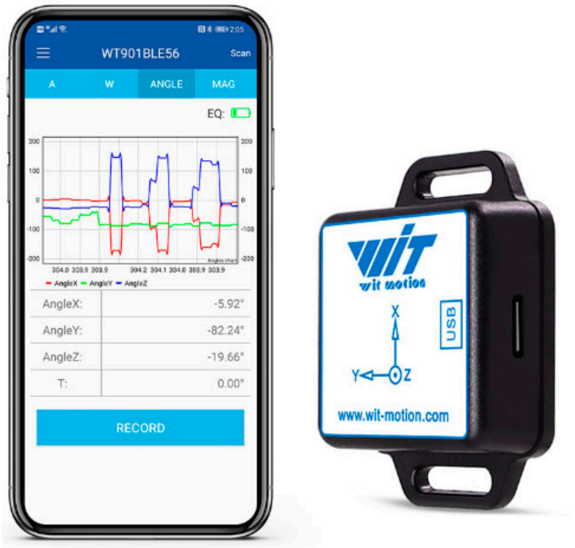

Supplementary Figure S3. Attitude sensor (WitMotion Bluetooth BLE 5.0 9 Axis Low-consumption Sensor WT901BLECL Angle + Acceleration + Gyro + Mag MPU9250 on PC/Android, China)

Soil moisture and measurement system

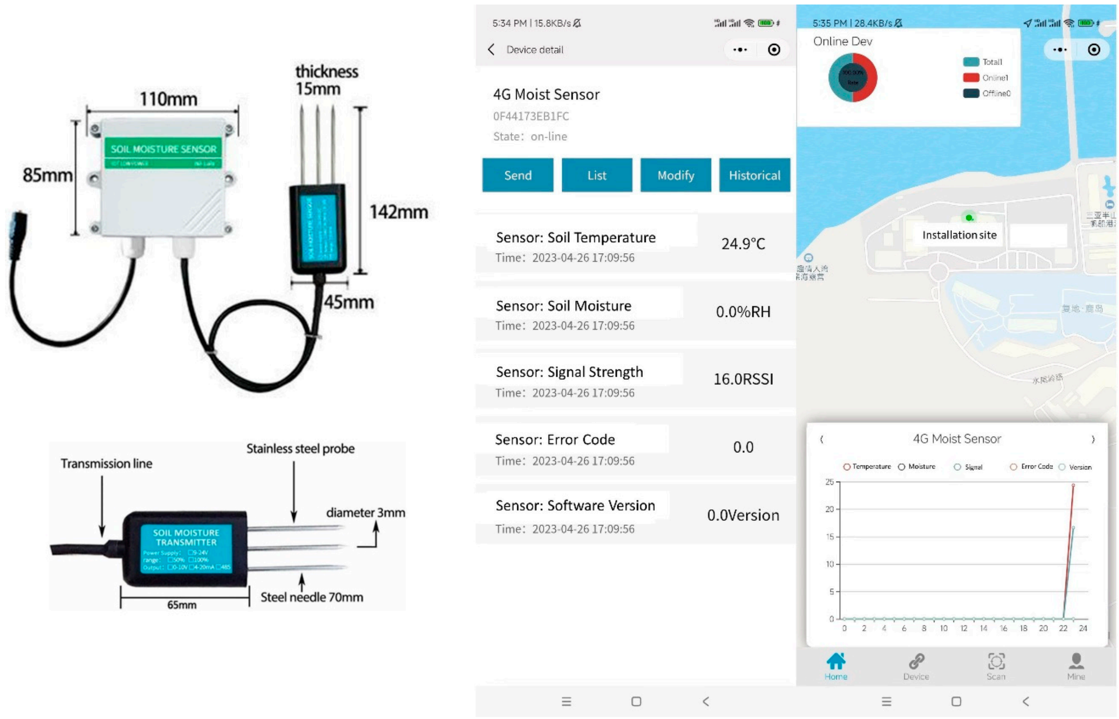

(a) (b)

Supplementary Figure S4. (a) JXBS-3001-TR_4G soil moisture sensor, (b) Sensor's wireless mobile app interface.

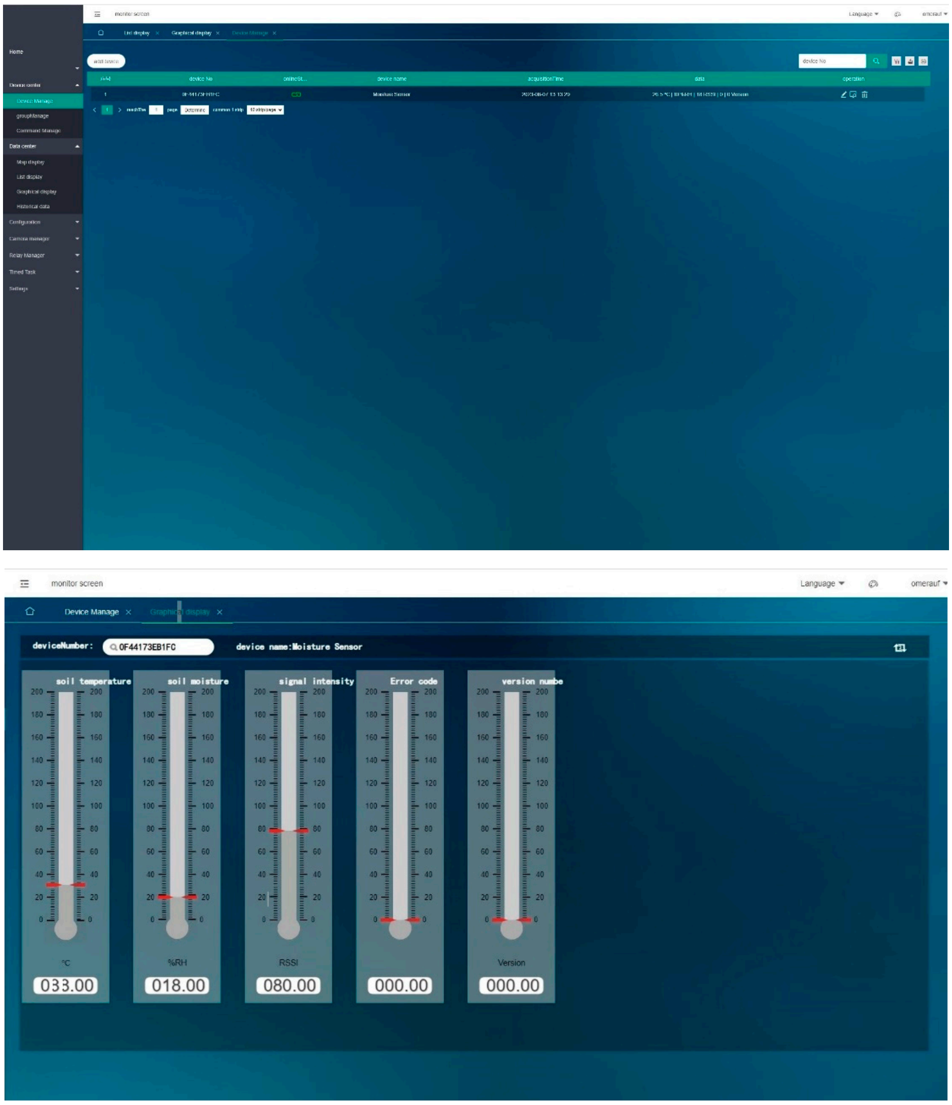

Supplementary Figure S5. Soil moisture sensors' real-time data monitoring web-based interface tool shows temperature, humidity, and signal strength.

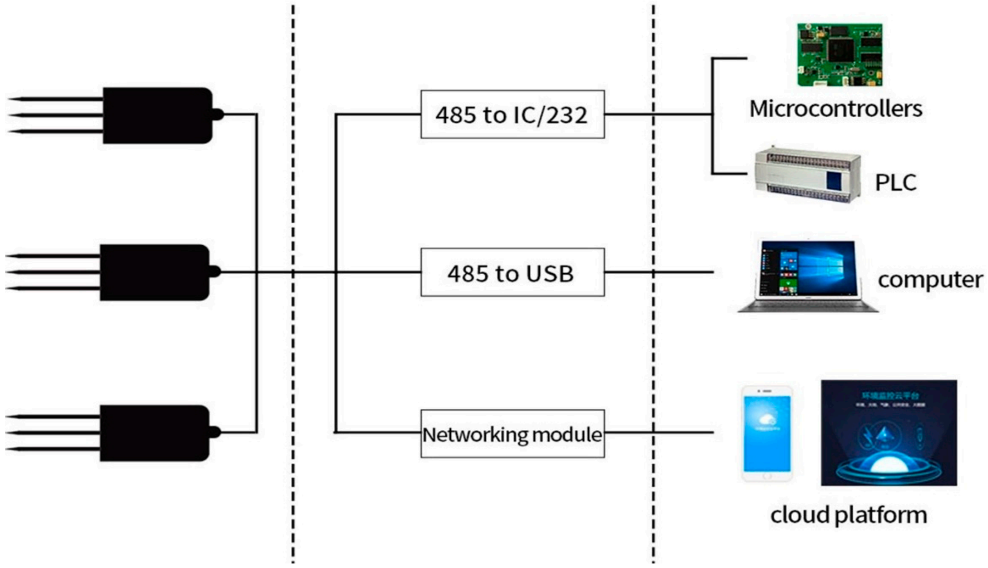

Supplementary Figure S6. JXBS-3001-TR_4G soil moisture sensor 4G wireless soil temperature and moisture sensor’s wireless communication modules.

Data acquisition FD0843 (6 Channel)

The FD0843, with its integrated Wireless Serial Communication Module, serves as a data collection hub, adept at gathering and transmitting information from sensors through its 6 channels. When pressure sensors are connected to the FD0843, each sensor's data flows into one of these channels, simplifying data aggregation and transmission. Its wireless capability enhances the ease and versatility of data retrieval, facilitating both real-time monitoring and subsequent analysis on a computer. By analyzing this data, we can gain deeper insights into how different loads impact sinkage, enriching our knowledge of pressure sinkage behaviour. To ensure precision in data collection, it's essential to install, set up, and calibrate both the FD0843 and the pressure sensors correctly. For optimal setup and operation, always adhere to the manufacturer's instructions or seek advice from technical specialists.

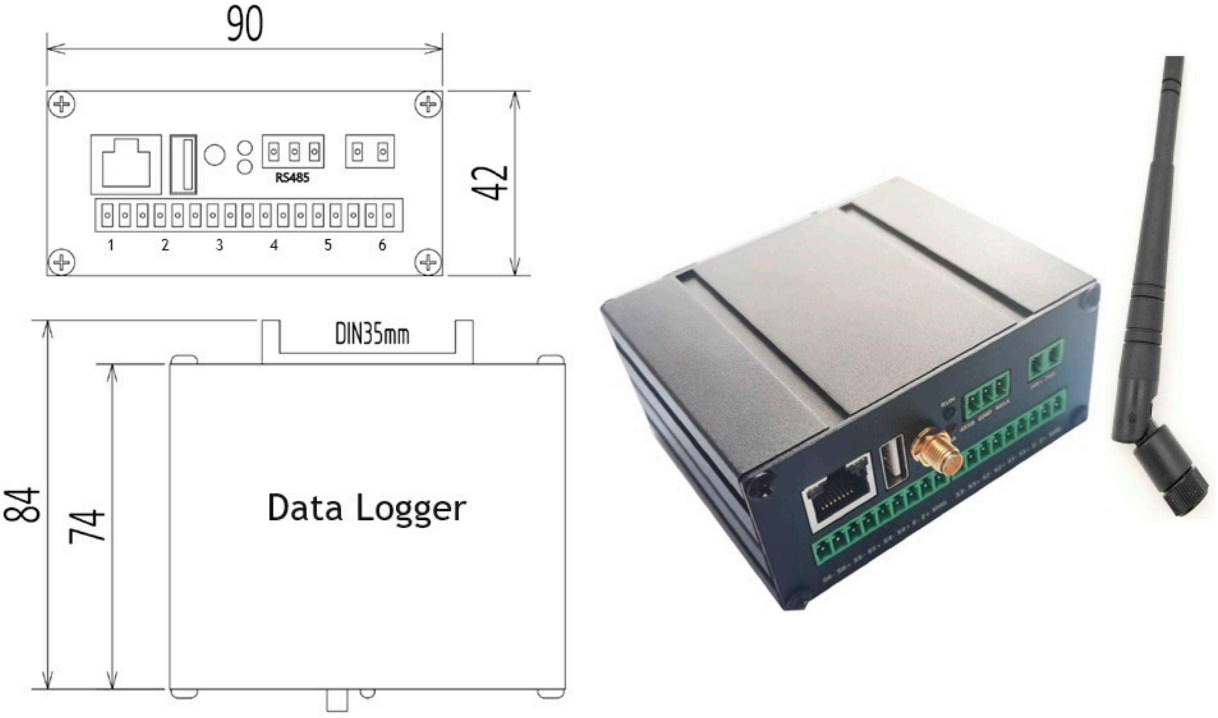

Supplementary Figure S7. 6-channel Wireless Data Acquisition System.

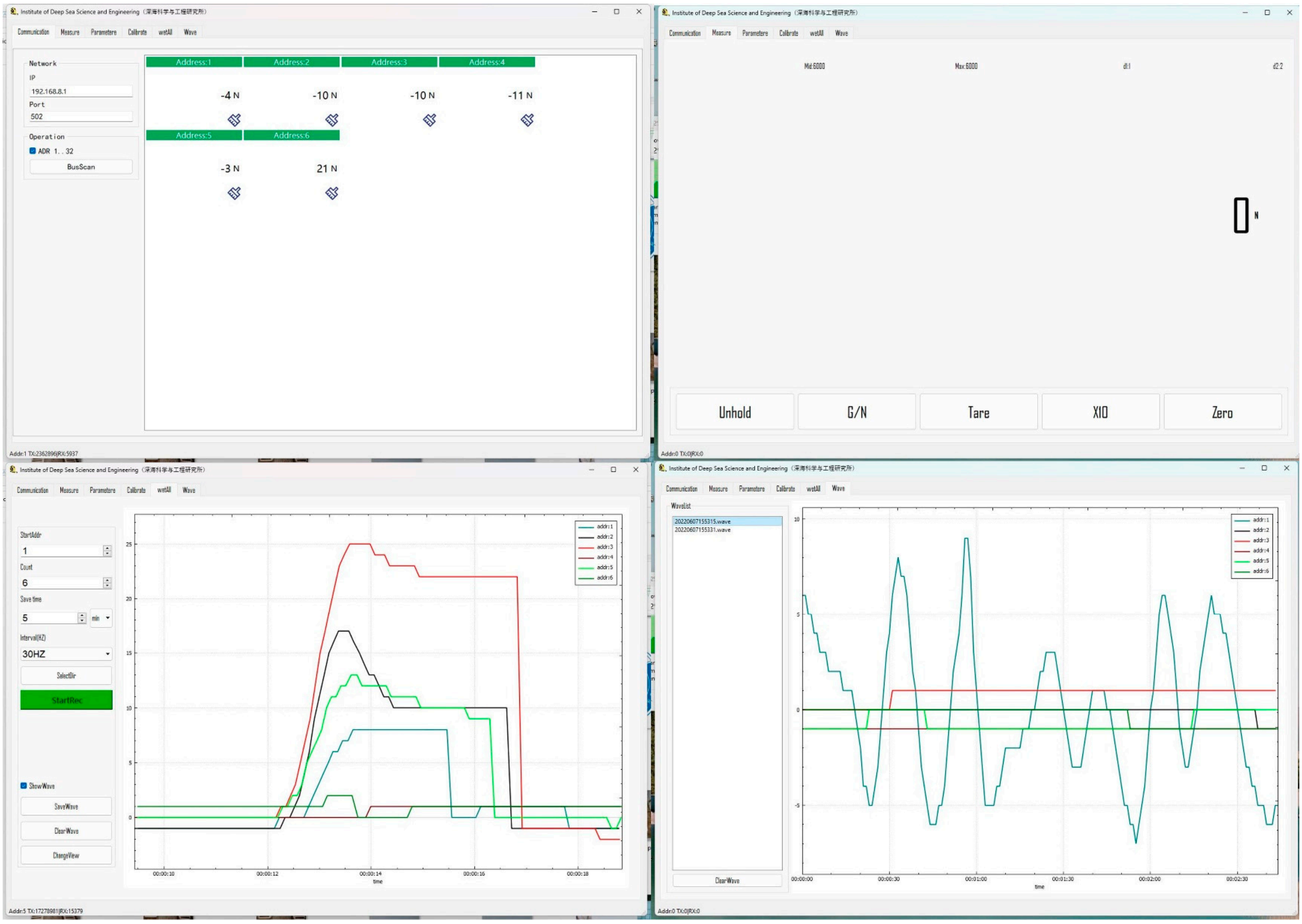

Supplementary Figure S8. 6-Channel Computer-Enabled Ground Pressure Measurement Software.

Data logger specifications

Supplementary Table S1. Data logger detailed specifications.

Parameters	Specifications
Body	Aluminum alloy housing
Size	90mm×42mm×84mm
Use environment	Temperature: －30° ～ 65 ℃;<br>Relative humidity: 10% ～ 95%
Protection	IP20
Operating Voltage	9～24 VDC, Power ＜10W
Supporting sensor	1～6
Maximum input signal	5mV/V
A/D update rate	1600Hz
Measurement rate	100/200/400/800/1600
Data transfer rate	100Mb/s
Sensor excitation voltage	5VDC
Minimum input sensitivity	0.5 μ V/d
Serial port	RS-485, 4800 ～ 115, 200 Baud rate
Wireless transmission	WiFi communication
Software	Computer-based software
Supporting protocol	MODBUS-RTU、MODBUS-UDP、Ether-CAT
Interface type	Ethernet

Soil Properties

Supplementary Table S2. Mechanical Soil Properties for Bentonite and Diatom Soils

Mechanical Soil Property	Bentonite	Diatom
Compressibility	High	Low
Shear Strength	Low	High
Friction Angle	Low	High
Cohesion	High	Low
Dilatancy	High	Low
Plasticity	High	Low

Loose Soil Properties

Supplementary Table S3. Loose Soil Properties for the Tracked Vehicle, Soil Bin Experiment

Parameter	Unit	Bentonite			Diatom		
Condition	-	Loose	Loose	Loose	Loose	Loose	Loose
Moisture Content (±)	%	10	20	30	10	20	30
Normal Stiffness	kPa	26	35	32	43	67.7	78
Terrain Stiffness (kØ)	kN/m	18	17.1	20	27	32	37
Ground Pressure	PSI	23	28	32	28	32	37
Sinkage	cm	2.5	3.6	4.8	1.8	2.6	3.8
Sinkage Ratio (s/D)	-	0.19	0.18	0.16	0.13	0.1	0.8
Shear Stiffness	kPa	12	17	19	26	31	37
Cohesion	kPa	6.5	8.7	10.5	4.2	5.5	6.3
Friction Angle	°	26	28.5	30	33	34.5	37
Hardening Ratio	-	1.7	2.1	2.32	1.5	2.15	2.6
Dilation Angle	°	6	5.5	6.5	3.5	5	6.5
Fluid Content	%	10	15	20	12	16	19
Ground Pressure	kPa	23	29	34	36	42	46

Compact Soil Properties

Supplementary Table S4. Compact Soil Properties for the Tracked Vehicle, Soil Bin Experiment

Parameter	Unit	Bentonite			Diatom		
Condition	-	Compact	Compact	Compact	Compact	Compact	Compact
Moisture Content (±)	%	10	20	30	10	20	30
Normal Stiffness	kPa	37	46	61	58	84	91
Terrain Stiffness (k∅)	kN/m	16.3	18.9	26	35	43	46
Ground Pressure	PSI	26	31	37	33	37	42
Sinkage	cm	1.1	2.3	3.1	0.8	1.3	2.1
Sinkage Ratio (s/D)	-	0.11	0.09	0.06	0.12	0.07	0.06
Shear Stiffness	kPa	16	19	26	37	41.6	48.5
Cohesion	kPa	8.5	12.7	14.5	7.3	8.2	9.5
Friction Angle	°	28	31	35	35	37	41
Hardening Ratio	-	2.3	2.5	2.7	2.6	3.3	3.8
Dilation Angle	°	7	7.9	8.8	6	7.5	8.7
Friction Coefficient	μ	0.8	0.65	0.58	0.9	0.84	0.76
Ground Pressure	kPa	31	38	45	33	46	52

Mechanics of track soil interaction

Supplementary Table S5. The Physical Properties of Bentonite, Diatom, and Sand Gravel Mixture

Parameter	Unit	Bentonite	Diatom	Sand Gravel mixture
Classification	-	Clay soil	Sedimentary deposit/rock	Silty gravel
Bulk density	g/cm^3	1.4 (Loose)<br>1.5 (Compact)	1.1 (Loose)<br>1.2 (Compact)	1.6 (Loose)<br>1.7 (Compact)
Water Moisture content	%	29	23	11
Internal friction angle	°	21	27	35
Cohesion	kPa	High	Low	Very low
Porosity	%	54	62	31
Swelling Capacity	%	High	Low	Very low
Specific gravity	-	2.4	2.2	2.6
Soil cone index (dry)	MPa	1.7	1.0	3.4
Soil cone index (wet)	MPa	1.1	0.6	1.9

Soil bin

Supplementary Table S6. The Parameters for the Soil Bin are Used for Pressure Sinkage and Bearing Capacity

Soil Bin Specifications	Values
Length	610 cm
Width	245 cm
Height	180 cm
Thickness	0.4 cm
Depth	175 cm
Material	Steel
Capacity	Adequate for accommodating small to medium rubber-tracked vehicles
Drainage System	Installed to control surplus water and soil saturation efficiently.
Side Walls	Smooth and vertical to reduce vehicle motion interference
Floor Surface	Even and level for uniform testing
Lighting	Adequate lighting for assessing visibility
Safety Features	Guardrails or obstacles for operator safety
Data Collection	Integration of sensor and data acquisition equipment

Soil's Detailed Properties

Supplementary Table S7. Detailed Properties of Test Soils

Parameter name	Unit	Value
Soil particle density		
• Bentonite	g/cm^3	2.7
• Diatom		2.3
• Sand gravel mixture		2.7
Natural moisture content		
• Bentonite	%	21
• Diatom		8
• Sand gravel mixture		9
Grain size distribution		
• Bentonite	mm	< 0.002
• Diatom		0.1
• Sand gravel mixture		2 ~ 5
Maximum dry density		
• Bentonite	g/cm^3	1.2
• Diatom		1.7
• Sand gravel mixture		1.8
Optimum moisture content		
• Bentonite	%	19
• Diatom		13
• Sand gravel mixture		8

Tracked Vehicle Motion Test

Supplementary Table S8. Rubber Tracked Vehicle Motion Test on Diatom and Bentonite Soil

Soil Type	Soil Density	Speed (m/sec)	Motion Observation
Bentonite soil	Loose (g/cm^3)	0.1	Very high sinkage, unable to move
Bentonite soil	Loose (g/cm^3)	0.2	N/A, unable to achieve speed
Bentonite soil	Loose (g/cm^3)	0.3	N/A, unable to achieve speed
Bentonite soil	Compacted (g/cm^3)	0.1	Moderate sinkage, able to move
Bentonite soil	Compacted (g/cm^3)	0.2	High sinkage, difficulty moving
Bentonite soil	Compacted (g/cm^3)	0.3	Unable to move, gets stuck
Diatom soil	Loose (g/cm^3)	0.1	High sinkage, low traction
Diatom soil	Loose (g/cm^3)	0.2	Higher sinkage, difficulty moving
Diatom soil	Loose (g/cm^3)	0.3	Unable to move, gets stuck
Diatom soil	Compacted (g/cm^3)	0.1	Low sinkage, good traction
Diatom soil	Compacted (g/cm^3)	0.2	Moderate sinkage, able to move
Diatom soil	Compacted (g/cm^3)	0.3	High sinkage, difficulty moving

Cone index measurement

Supplementary Table S9. Experimental Soil Properties and Cone Index Measurement for Bentonite and Diatom

Soil type	Moisture content ± %	Loose Density 3g/cm³	Compacted Density g/cm³	Compaction %	Depth cm	Cone Index kPa
Bentonite	10	1.3	1.5	15.38	10	94.12
					20	215.25
					30	127.36
					40	179.48
Bentonite	20	1.2	1.4	17.67	10	184.16
					20	94.28
					30	179.4
					40	127.52
Bentonite	30	1.1	1.3	18.18	10	94.44
					20	162.36
					30	127.6
					40	215.72
Diatom	10	0.9	1.1	22.29	10	269.14
					20	293.62
					30	318.11
					40	342.59
Diatom	20	0.8	1.0	25.4	10	219.38
					20	238.12
					30	257.86
					40	278.59
Diatom	30	0.7	0.9	28.57	10	175.21
					20	192.44
					30	212.67
					40	231.89
